# A cross-sectional study of the effects of load carriage on running characteristics and tibial mechanical stress: implications for stress-fracture injuries in women

**DOI:** 10.1186/s12891-017-1481-9

**Published:** 2017-03-23

**Authors:** Chun Xu, Amy Silder, Ju Zhang, Jaques Reifman, Ginu Unnikrishnan

**Affiliations:** 1 Department of Defense Biotechnology High Performance Computing Software Applications Institute, Telemedicine and Advanced Technology Research Center, United States Army Medical Research and Materiel Command, Fort Detrick, Maryland USA; 2 Department of Bioengineering, Stanford University, Stanford, California USA; 3 Auckland Bioengineering Institute, University of Auckland, Auckland, New Zealand

**Keywords:** Stress fracture, Tibial mechanical stress, Finite element analysis, Running mechanics, Load carriage

## Abstract

**Background:**

Load carriage is associated with musculoskeletal injuries, such as stress fractures, during military basic combat training. By investigating the influence of load carriage during exercises on the kinematics and kinetics of the body and on the biomechanical responses of bones, such as the tibia, we can quantify the role of load carriage on bone health.

**Methods:**

We conducted a cross-sectional study using an integrated musculoskeletal-finite-element model to analyze how the amount of load carriage in women affected the kinematics and kinetics of the body, as well as the tibial mechanical stress during running. We also compared the biomechanics of walking (studied previously) and running under various load-carriage conditions.

**Results:**

We observed substantial changes in both hip kinematics and kinetics during running when subjects carried a load. Relative to those observed during running without load, the joint reaction forces at the hip increased by an average of 49.1% body weight when subjects carried a load that was 30% of their body weight (ankle, 4.8%; knee, 20.6%). These results indicate that the hip extensor muscles in women are the main power generators when running with load carriage. When comparing running with walking, finite element analysis revealed that the peak tibial stress during running (tension, 90.6 MPa; compression, 136.2 MPa) was more than three times as great as that during walking (tension, 24.1 MPa; compression, 40.3 MPa), whereas the cumulative stress within one stride did not differ substantially between running (15.2 MPa · s) and walking (13.6 MPa · s).

**Conclusions:**

Our findings highlight the critical role of hip extensor muscles and their potential injury in women when running with load carriage. More importantly, our results underscore the need to incorporate the cumulative effect of mechanical stress when evaluating injury risk under various exercise conditions. The results from our study help to elucidate the mechanisms of stress fracture in women.

## Background

The frequent occurrence of stress-fracture injuries during basic combat training (BCT) remains a leading concern for the United States (US) military [1]. Epidemiological studies have shown that the most commonly injured sites for stress fractures include the tibia, femoral neck, tarsal navicular, metatarsals, and pelvis [2–4]. Stress fractures are caused by repeated application of sub-critical mechanical insults associated with common BCT exercises, including walking, running, and jumping [1, 5–7]. Furthermore, military personnel are often required to carry loads that reach a considerable percentage of their body weight, which have been shown to be associated with an increased risk of stress fracture [8, 9]. To understand the impact of load carriage and ultimately help reduce the risk of stress fracture, previous studies have examined systematic changes in metabolic parameters (e.g., energy cost) [10–12], ground reaction forces (GRFs) [13], joint kinematics [10, 14], and muscle activities [15] due to load carriage. However, the effects of load carriage on bone biomechanical responses (e.g., bone stress and strain), which play a crucial role in bone health, have been less extensively investigated.

The technical challenges and ethical restrictions associated with implanting strain gauges make it impractical to experimentally measure bone strains in healthy individuals [16]. Therefore, computational methods have been implemented in recent years to quantify mechanical bone stress and strain [17]. For example, our group has developed an integrated musculoskeletal-finite-element (FE) modeling framework and used it to quantify the impact of load carriage during walking in a woman [18] on a number of measures, including joint kinematics, joint kinetics (joint reaction forces or JRFs), and biomechanical responses of the tibia (i.e., spatiotemporal stress distribution, and its cumulative effect during one gait cycle).

Previous studies have shown that the muscles involved during walking and running are almost identical, and that switching from walking to running is associated with changes in both the timing and intensity of muscle activation [19]. However, our understanding of how the body adjusts its kinetics, kinematics, and biomechanics over a range of load carriages during running remains fragmentary. Here, we sought to answer the following research question: how do the joint angles, JRFs, and tibial mechanical stresses change in response to load carriage when women run at their preferred speeds? We hypothesized that: *1*) running with load carriage would increase both peak JRFs and the percentage of the tibial volume subjected to high stress and *2*) the changes in JRFs and tibial stress would not be proportional to the change in load carriage.

## Methods

**Fig. 1:**
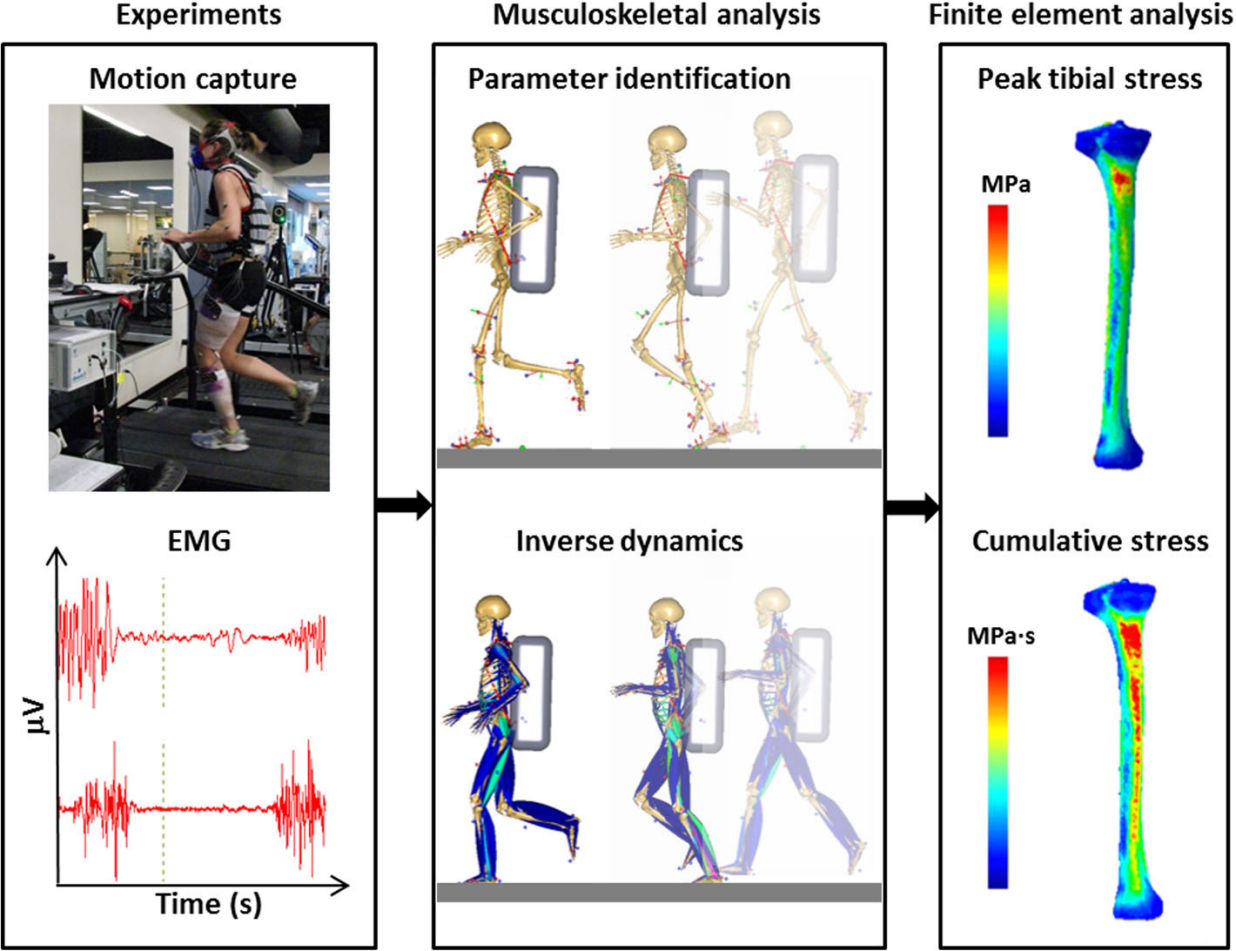
Flowchart of the integrated musculoskeletal-finite-element modeling method. EMG: electromyography

We performed musculoskeletal-FE analyses by using a recently reported method [18]. Figure 1 shows an overview of our integrated approach.

### Experiments

Our musculoskeletal analyses were based on data obtained from motion-capture experiments described by Silder et al. [20]. Briefly, four female recreational runners [age, 29.8 yr (95% CI: 27.8, 31.7); mass, 60.0 kg (55.8, 64.3); height: 1.68 m (1.66, 1.71); body mass index (BMI): 21.2 kg/m^2^ (19.8, 22.6)] ran on a force-sensing treadmill (Bertec Corporation, Columbus, OH, USA), at their preferred speeds without carrying any load (0% baseline model) or carrying a load of 20% or 30% of their body weight (BW) by using an adjustable weight vest. For each participant, 29 retroreflective markers were secured on specific anatomical locations, including the clavicle bone, arms, trunk, pelvis, thighs, shanks, and feet. All subjects were free of injury (current or past) and signed a written consent form approved by the Stanford University Institutional Review Board. The output from the motion-capture experiments included marker trajectories, GRFs, and electromyography (EMG) recordings.

### Musculoskeletal analysis

We used the AnyBody Modeling System^TM^ (AnyBody Technology, Aalborg, Denmark) to perform musculoskeletal analyses based on the motion-capture data. We first filtered the marker trajectories and GRF recordings with a low-pass fourth-order Butterworth filter (cutoff frequency, 8 Hz). In the absence of subject-specific anatomical and musculoskeletal parameters, we used the anthropometric data to scale a previously validated whole-body musculoskeletal model [21]. The lower extremities of the musculoskeletal model consisted of seven rigid bodies, including the pelvis, thigh, shank, and foot, as well as 55 Hill-type muscles per leg [22]. We modeled the hip joints as spherical joints (enabling flexion/extension, internal/external rotation, and abduction/adduction movements), and the knee and ankle joints as revolute joints (allowing flexion/extension rotation). A revolute joint connected the patella to the femur, which allowed a small degree of rotation but no translation. A patellar tendon connected the patella segment to the tibia.

One common challenge associated with musculoskeletal modeling based on motion-capture experiments is determining the length of each body segment [23]. This is because the joints are located deep below the skin and the tissues, making it difficult to measure their true positions without advanced imaging techniques. Therefore, we first obtained the joint positions and orientations by using a least-squares parameter identification algorithm that minimizes the errors between markers defined in the model and those tracked in the experiment [23]. We used an inverse dynamics approach to compute muscle activities and JRFs over one running cycle, where muscle activity was defined as the muscle force divided by muscle strength [21]. We performed whole-body musculoskeletal analysis for running without a load (0%), or with a load of 20% or 30% BW for all four subjects. We normalized the moments and JRFs for each participant by the body mass to enable consistent comparisons.

### FE analysis

Details of the FE analysis method have been reported previously [18]. Briefly, we first generated a tetrahedral FE mesh from computed tomography images of a sex-, age-, and BMI-matched subject from the Victorian Institute of Forensic Medicine (Melbourne, Victoria, Australia) database. Subsequently, we used a mesh-morphing and material-property-mapping algorithm to derive heterogeneous bone material properties for each tibia model of interest [24, 25]. This procedure produced an average of 147,357 unique linear elastic and isotropic material properties for the tibia model. Finally, we exported the material property definitions into an ABAQUS input file for structural analysis (ABAQUS 6.12, Dassault Systèmes, Vélizy-Villacoublay, France).

We used the muscle forces and bone forces/moments obtained from the musculoskeletal analysis to specify the loading conditions for the FE analysis. Specifically, we first identified muscle/ligament insertion points from the musculoskeletal model to construct FE constraint nodes, which were then coupled to the outer surface of the tibial FE meshes. Through this procedure, we specified an average of 171 couplings between the muscle/ligament nodes and the tibial FE meshes. We defined the joint contact forces in a similar manner. Finally, we performed mesh convergence studies, using the FE loading conditions defined for the baseline model. We determined 3.5 mm as an adequate edge-length for our 10-node quadratic tetrahedral mesh.

From the FE analyses, we determined the spatiotemporal tibial stress distribution, and then evaluated the per-cycle cumulative effects of tibial stress (MPa · s) by integrating the FE-predicted nodal stress values over time [26]. In the absence of any experimentally measured threshold above which the tibial mechanical stress becomes harmful, we first determined three stress thresholds from the baseline model that divided the tibia into four zones of equal volume (i.e., 25% of the total volume). Then, we calculated the changes in volumetric fraction at load carriage conditions of 20% and 30% BW according to the determined stress thresholds. Lastly, we quantified the changes in the volumetric fraction of cumulative stress due to load carriage in a similar manner.

## Results

### Muscle activities

**Fig. 2:**
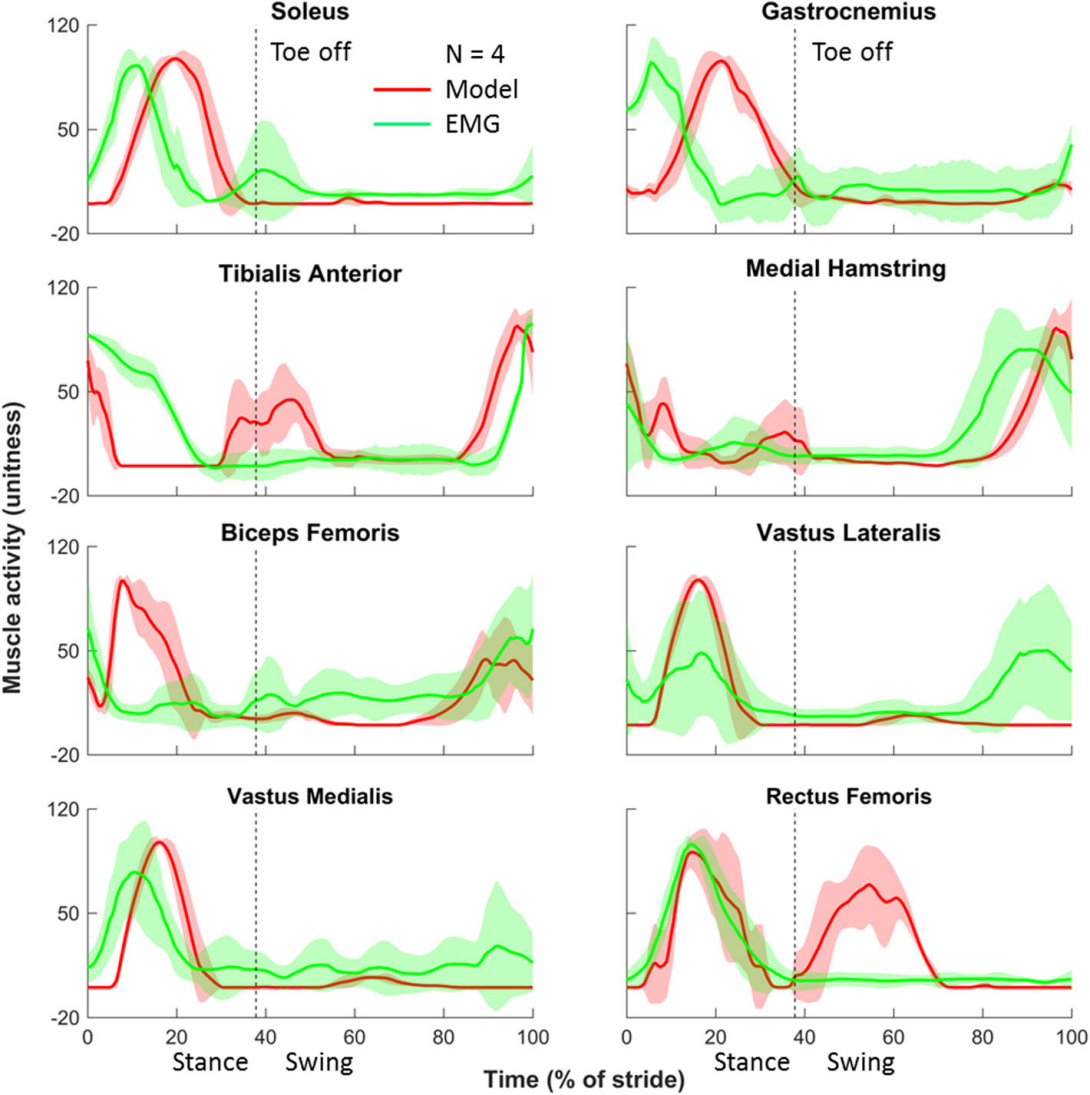
Predicted and measured muscle activities without load carriage. *Red* and *green lines* show the muscle activities predicted by the model and those measured by electromyography (EMG), respectively, as a function of the percentage of stride. We normalized the magnitudes by the maximal values for each curve. The solid lines represent the group averages of simulated and measured muscle activities (*N* = 4). The shaded areas represent the group-averaged EMG linear envelopes (mean ± one standard deviation). The vertical dashed line, which represents the toe-off point for each condition, separates the stance phase (before toe off) from the swing phase (after toe off)

To ensure the validity of the musculoskeletal model—that it correctly predicted the muscle activation needed to produce the measured whole-body kinematics—we compared the time courses of the predicted muscle forces and EMG recordings under baseline conditions [27]. We observed good agreement between the predicted and measured muscle activity profiles under the baseline condition after taking into account the electromechanical delay, which represents the time lag between the onset of muscle activation (measured by EMG) and muscle force generation (predicted by the model) [28] (Fig. 2: red, model predictions; green, EMG measurements). Specifically, the model correctly predicted activation of the ankle plantarflexor muscles (soleus and gastrocnemius) during mid-stance and of the ankle dorsiflexor muscles (tibialis anterior) during the late-swing and early-stance phases. Consistent with the EMG recordings, the model predicted medial hamstring activation in the latter half of the swing phase. The model also predicted peak biceps femoris activation during the early stance phase, while the EMG recordings from this muscle peaked earlier (albeit at a lower level), at the end of the swing phase of the previous stride. The model predicted the onset of knee extensor (vastus lateralis, vastus medialis, and rectus femoris) activity at mid-stance to absorb the shock. In addition, the model predicted low muscle activity in the vastus lateralis throughout the swing phase, although two of four subjects’ EMG recordings showed muscle activities only during the late-swing phase. This indicates considerable inter-subject variability in the EMG recordings.

### Kinematics and kinetics

**Fig. 3:**
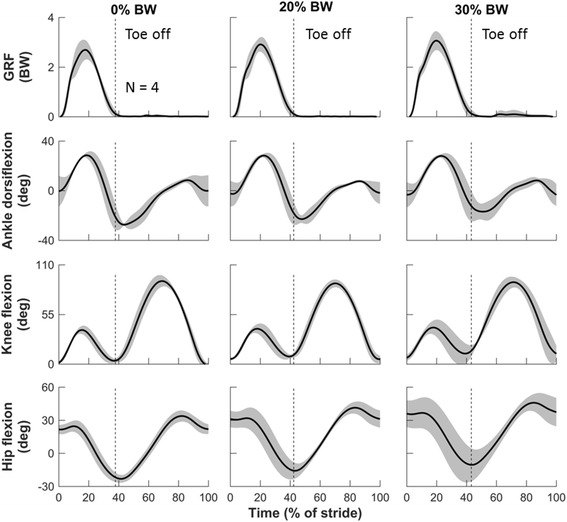
Joint kinematics at different loads during one gait cycle in the sagittal plane. Solid lines represent means of four subjects. Shaded areas represent one standard deviation above and below the means. For convenience, the first row shows ground reaction force (GRF). Each graph begins and ends at initial contact. The vertical dashed line, which represents the toe-off point for each condition, separates the stance phase (before toe off) from the swing phase (after toe off). Positive angles represent flexion, and negative values represent extension. BW: body weight; deg: degree

**Fig. 4:**
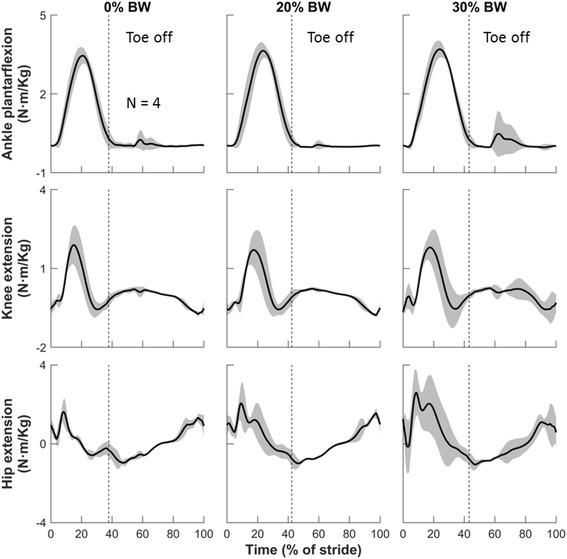
Joint moments at different loads during one gait cycle in the sagittal plane. Solid lines represent means of four subjects. Shaded areas represent one standard deviation above and below the means. Each graph begins and ends at initial contact. The vertical dashed line, which represents the toe-off point for each condition, separates the stance phase (before toe off) from the swing phase (after toe off). Results are normalized by body mass (in kg). Positive angles and moments represent extension, and negative values represent flexion. BW: body weight

**Fig. 5:**
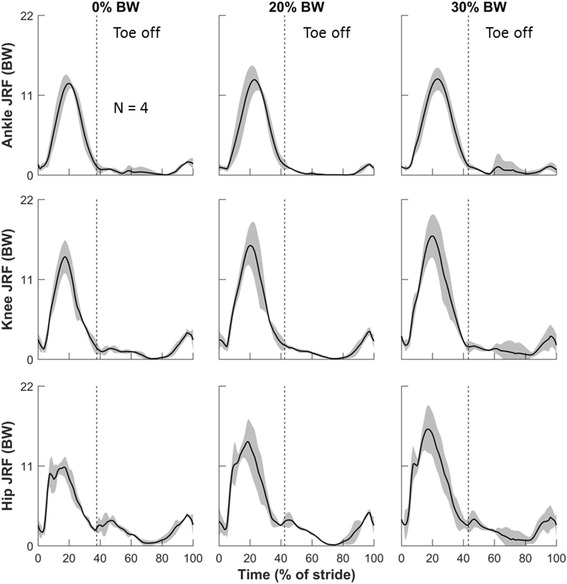
Joint reaction forces (JRFs) at different loads during one gait cycle. Solid lines represent means of four subjects. Shaded areas represent one standard deviation above and below the means. Each graph begins and ends at initial contact. The vertical dashed line, which represents the toe-off point for each condition, separates the stance phase (before toe off) from the swing phase (after toe off). Results are shown as normalized dimensionless quantities. BW: body weight

All participants adopted a rear-foot-strike running style except one subject, who was a forefoot-strike runner. Their running speeds were 3.4, 3.0, 3.5, and 3.6 m/s. Figures 3, 4 and 5 depict the means and SDs of the kinematic (Fig. 3) and kinetic (Figs. 4 and 5) parameters at each joint during running with and without load carriage; their peak values are summarized in Table 1. Consistent with the results of a previous study [20], load carriage increased the stance time and magnitude of the GRFs. Specifically, Table 1 shows that carrying a 30% BW load increased the stance from 37.8% (SD = 1.1) in the baseline condition to 43.2% (SD =1.8) of a gait cycle (i.e., an average increase of 14.3%). The peak vertical GRF increased from 2.7 BW (SD = 0.4) in the baseline condition to 3.1 BW (SD = 0.4) for 30% BW load carriage (i.e., an average increase of 14.8%; Fig. 3, Table 1).

**Table 1:** Mean and standard deviation of peak joint angles, joint moments, and joint reaction forces while running without a load (0%) or with an additional load of 20% or 30% of body weight (BW) (*N* = 4)

	Load carriage (% BW)		
	0	20	30
Stance duration (%)	37.8 (1.1)	42.3 (2.5)	43.2 (1.8)
Ground reaction force (BW)	2.7 (0.4)	2.9 (0.3)	3.1 (0.4)
Peak joint angles (degree)			
Ankle			
Dorsiflexion	28.5 (2.2)	28.2 (1.7)	27.9 (1.5)
Plantarflexion	27.4 (1.1)	22.9 (3.0)	17.9 (6.7)
Knee			
Flexion during stance phase	37.7 (4.5)	39.1 (5.2)	40.6 (7.9)
Flexion during swing phase	92.0 (6.3)	89.5 (4.3)	90.7 (5.9)
Hip			
Flexion	33.8 (5.1)	41.3 (5.5)	45.8 (7.9)
Extension	23.2 (3.5)	15.8 (7.3)	10.5 (14.8)
Adduction	5.3 (2.0)	5.8 (0.9)	5.0 (1.9)
External rotation	6.4 (2.4)	6.3 (3.5)	7.9 (2.7)
Peak joint moments (N · m/kg)			
Ankle			
Plantarflexion	3.4 (0.3)	3.6 (0.3)	3.7 (0.3)
Knee			
Flexion	0.7 (0.1)	0.8 (0.1)	0.7 (0.1)
Extension	1.9 (0.7)	1.7 (0.7)	1.8 (0.5)
Hip			
Flexion	1.0 (0.1)	1.0 (0.2)	1.1 (0.3)
Extension	1.6 (0.7)	2.1 (0.9)	2.6 (1.2)
Peak joint reaction forces (BW)			
Ankle	12.6 (0.8)	13.1 (1.7)	13.2 (1.6)
Knee	14.1 (2.3)	15.6 (2.9)	17.0 (3.0)
Hip	10.8 (1.4)	14.4 (3.0)	16.1 (3.2)

When running with a 30% BW load carriage, the peak ankle dorsiflexion and plantarflexion decreased from 28.5 deg (SD = 2.2) and 27.4 deg (SD = 1.1) in the baseline condition to 27.9 deg (SD = 1.5) and 17.9 deg (SD = 6.7) after toe off, respectively (i.e., an average decrease of 2.1% and 34.7%, respectively; Fig. 3, Table 1). The peak ankle plantarflexion moment increased from 3.4 N · m/kg (SD = 0.3) in the baseline condition to 3.7 N · m/kg (SD = 0.3) (i.e., an average increase of 8.8%; Fig. 4, Table 1). The peak ankle JRFs increased from 12.6 BW (SD = 0.8) in the baseline condition to 13.2 BW (SD = 1.6) (i.e., an average increase of 4.8%; Fig. 5, Table 1).

The knee was flexed throughout the entire running gait, with one flexion peak occurring near mid-stance and the other near mid-swing. During the absorption phase of the stance, the knee flexed to approximately 45 deg. This was followed by the maximal knee flexion during the swing phase, which reached approximately 90 deg (Fig. 3). We did not observe systematic changes in knee angles or knee moments as the load increased (Figs. 3 and 4, Table 1). When running without a load, the knee, among the lower-extremity structures examined, was exposed to the greatest JRFs, which reached as much as 14.1 BW (SD = 2.3) [ankle, 12.6 BW (SD = 0.8); hip, 10.8 BW (SD = 1.4)]. Running with a 30% BW load increased the knee JRFs to 17.0 BW (SD = 3.0), corresponding to a 20.6% increase in average peak knee JRFs (Fig. 5, Table 1).

Carrying a 30% BW load while running limited hip extension but increased hip flexion. The peak hip extension at toe off decreased from 23.2 deg (SD = 3.5) in the baseline condition to 10.5 deg (SD = 14.8) (i.e., an average decrease of 54.7%; Fig. 3, Table 1). All subjects leaned forward to compensate for the reduced hip extension, as evidenced by the increased peak hip flexion before the terminal swing phase from 33.8 deg (SD = 5.1) in the baseline condition to 45.8 deg (SD = 7.9) with a 30% BW load (i.e., an average increase of 35.5%; Fig. 3, Table 1). We did not observe systematic changes in hip adduction and external rotation in response to load carriage. The mean peak hip flexion and extension moment increased from 1.0 N · m/kg (SD = 0.1) and 1.6 N · m/kg (SD = 0.7) in the baseline condition to 1.1 N · m/kg (SD = 0.3) and 2.6 N · m/kg (SD = 1.2), respectively (i.e., an average increase of 10.0% and 62.5%, respectively; Fig. 4, Table 1). The mean peak hip JRFs increased from 10.8 BW (SD = 1.4) to 16.1 BW (SD = 3.2) (i.e., an average increase of 49.1%; Fig. 5, Table 1).

### Tibial stress and cumulative stress

**Fig. 6:**
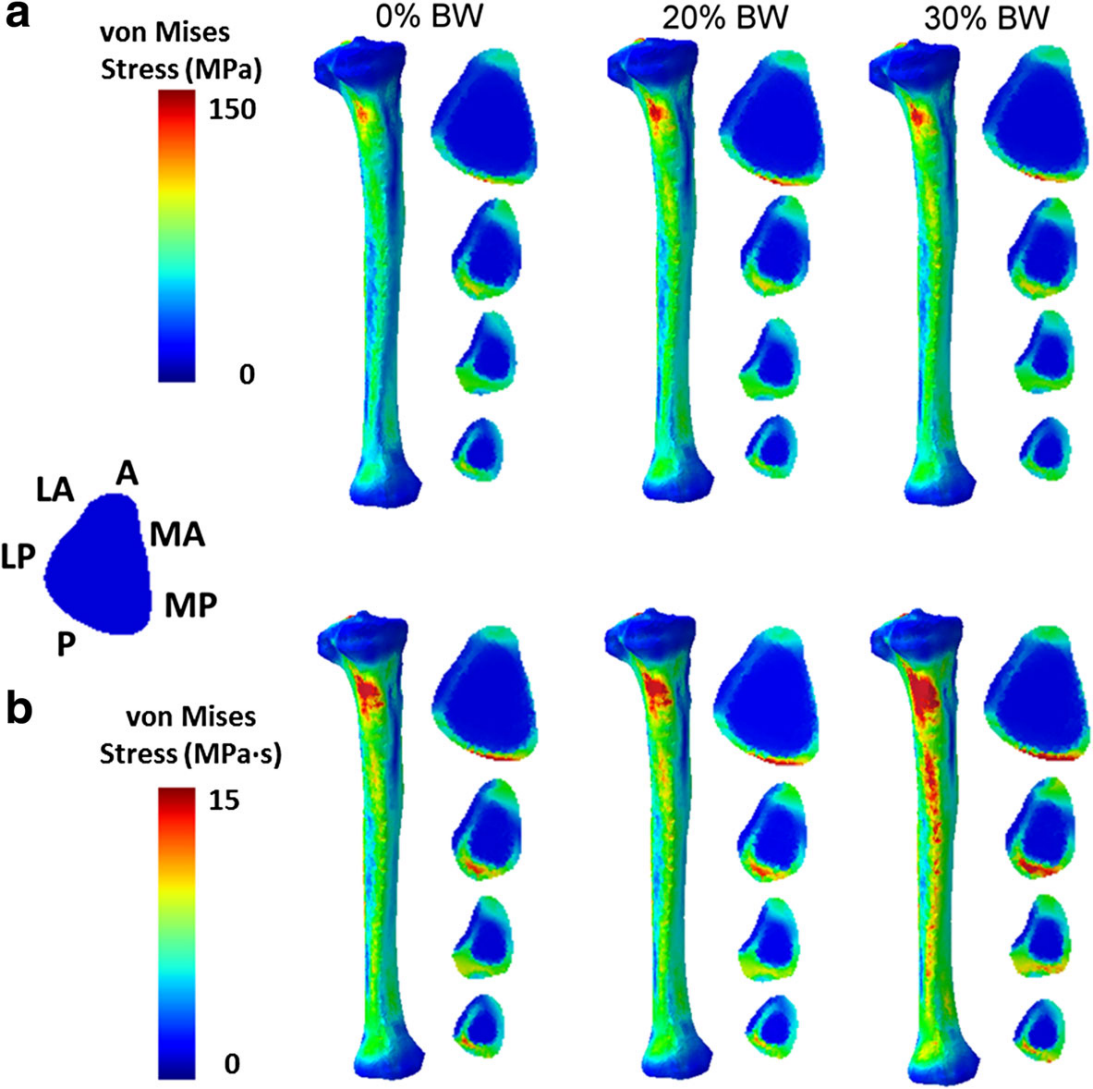
Spatiotemporal distribution of tibial stresses. Top and bottom panels show tibial von Mises stresses (**a**) and cumulative tibial stresses (**b**), respectively, during one gait cycle without a load (0% baseline, left), with a 20% body weight (BW) load (center), and a 30% BW load (right). We divided the cross section of the left tibia into six sectors. A: Anterior; MA: Medial Anterior; MP: Medial Posterior; P: Posterior; LP: Lateral Posterior; LA: Lateral Anterior. To emphasize the high-stress region on the medial-posterior side, the anterior-posterior (A-P) axis of the 3-D tibia is set oblique relative to the page. Tibial cross sections are arranged with A-P axis running vertically along the page

Figure 6 shows von Mises stress distributions in the tibia of one subject when running without a load, with a 20% BW load, and with a 30% BW load. For all subjects, the load-related changes in von Mises stress (Fig. 6a) were less pronounced than those in cumulative stress (Fig. 6b). The observed high-stress regions were mainly on the medial-posterior side of the upper diaphysis, corresponding to the location where the semitendinosus muscle of the hamstring inserted, and the posterior side of the mid-diaphysis, corresponding to the location of the superficial posterior muscle compartment, including the gastrocnemius and soleus muscles. Similar to our previous findings of load carriage during walking, the anterior crest of the tibia was exposed to tension, and the posterior aspect subjected to compression [18]. However, the peak values of tibial von Mises stress appeared to be unaffected by load carriage (Table 2).

**Table 2:** Mean and standard deviation of model-predicted peak tibial von Mises stresses during running without a load (0%), or with an additional load of 20% or 30% of body weight (BW) (*N* = 4)

Load carriage (% BW)	Tension (MPa)	Compression (MPa)
0	101.2 (10.7)	146.3 (8.9)
20	103.7 (17.7)	145.3 (10.1)
30	102.3 (26.0)	147.1 (19.9)

**Fig. 7:**
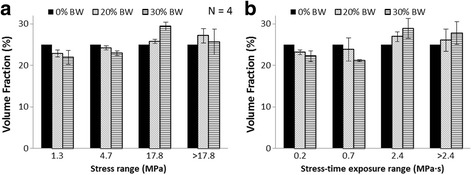
Histograms of tibial volume fractions subjected to different stress levels. Left and right panels show the tibial volume fractions subjected to different levels of stress (**a**) and cumulative stress (**b**), respectively, as a function of carried load. BW: body weight

In the baseline model, we identified pseudo-thresholds in the peak von Mises stress distributions [1.3 MPa (SD = 0.0), 4.6 MPa (SD = 0.2), and 17.8 MPa (SD = 1.4)] and cumulative stress distributions [0.2 MPa · s (SD = 0.0), 0.7 MPa · s (SD = 0.1), and 2.4 MPa · s (SD = 0.1)] for the three load conditions. Histograms of the tibial stress distribution indicated that the average percentage of the tibia exposed to stresses between 4.7 MPa and 17.8 MPa increased from 25.0% at baseline to 25.8% or 29.4% when carrying a 20% or 30% BW load, respectively, while the fraction of the tibia subjected to stresses greater than 17.8 MPa increased from 25.0% at baseline to 27.2% or 25.7% when carrying a 20% or 30% BW load, respectively (Fig. 7a). The average percentage of the tibia exposed to cumulative stresses between 0.7 MPa · s and 2.4 MPa · s increased from 25.0% at baseline to 26.9% or 28.9% when carrying a 20% or 30% BW load, respectively, while the fraction of the tibia subjected to cumulative stresses greater than 2.4 MPa · s increased from 25.0% at baseline to 26.1% or 27.8% when the subject carried a load of 20% or 30% of BW, respectively (Fig. 7b). Consequently, the tibial volume subjected to “high” stress (>4.7 MPa) increased from 50.0% in the baseline running condition to 53.0% (= 25.8% + 27.2%) and 55.1% (= 29.4% + 25.7%) when carrying 20% and 30% BW loads, respectively (Fig. 7a). Likewise, the tibial volume subjected to “high” cumulative stress (> 0.7 MPa · s) increased from 50.0% in the baseline running condition to 53.0% (= 26.9% + 26.1%) and 56.7% (= 28.9% + 27.8%) when carrying 20% and 30% BW loads, respectively (Fig. 7b).

## Discussion

We performed integrated musculoskeletal-FE simulations to emulate the effects of load carriage on joint kinematics, joint kinetics, tibial mechanical stress, and cumulative tibial stress per gait cycle in four women running at their preferred speeds. We chose the tibia as the bone of interest because it is the most frequently injured site in athletes [29–31] and military recruits [32]. We chose female subjects because of the growing number of women serving in the Army [33]. As are their male counterparts, women are required to wear body armor and carry heavy loads averaging 45.5 kg [34]. Consequently, they are at a higher risk for developing stress-fracture injuries than are men [33].

In support of our first hypothesis, both the peak JRFs and the percentage of the tibial volume subjected to high stresses and cumulative stresses per cycle increased with an increase in load carriage. It is noteworthy that the fraction of tibial volume subjected to pre-defined stress thresholds changed in response to load carriage in a non-monotonic manner. Specifically, carrying a 30% BW load increased the average peak JRFs of the ankle, knee, and hip by 4.8%, 20.6%, and 49.1%, respectively, when compared with the baseline condition (Table 1). The tibial volume subjected to stresses between 4.7 MPa and 17.8 MPa increased from 25.0% for the baseline condition to 25.8% for a 20% BW and 29.4% for a 30% BW load carriage, whereas that subjected to stresses greater than17.8 MPa increased from 25.0% for the baseline condition to 27.2% for 20% BW but only to 25.7% for 30% BW load carriage. This might be the result of the quadratic muscle-recruitment criterion adopted in the model, because such a mechanism, which tends to recruit more muscles during high-intensity tasks, distributes the load among several muscles spanning the joint to prevent a few muscles from being overloaded and consequently undergoing muscle fatigue [21]. Overall, the percentage changes in the JRFs, tibial peak stress, and cumulative stress were disproportional to that of the load carriage, consistent with our second hypothesis.

Our results showed that both the kinematics and kinetics of the hip changed substantially during running with load carriage. When compared with the baseline condition, carrying a 30% BW load decreased the mean peak hip extension at toe off by 54.7% and increased the mean peak hip flexion and extension moments by 10.0% and 62.5%, respectively, in addition to increasing the mean peak hip JRFs (49.1%, Table 1). These results highlight the importance of hip muscles in high-intensity physical activity (e.g., running with load carriage), and are supported by previous studies linking them with sports injuries. For example, in collegiate female athletes with patellofemoral injury, hip abductors and external rotators are significantly weaker in the injured leg than in the unaffected leg [35]. Furthermore, if the hip extensors do not generate enough power, either other muscles in the lower extremities will compensate for the reduced force, or movement patterns will change, which in turn may result in increased energy costs and early muscle fatigue [36]. Such changes may be related to a number of biomechanical alterations that potentially increase the risk of impact-related injuries in the lower extremities (e.g., Achilles tendinosis, patellofemoral dysfunction, and tibial stress fracture) [37]. Preparatory strength and endurance training exercises before military BCT, such as squats, incline sit-ups, leg raises, and interval training, which are designed to strengthen hip flexor muscles and pelvic-stabilizing muscles (gluteus medius), are effective in assisting prospective Service members to endure BCT without injuries [38].

Consistent with telemeterized bilateral joint-replacement studies [39, 40], our findings suggest that, for the subject who completed both walking [18] and running experiments, the peak running JRFs were more than two times greater than the peak walking JRFs, with the contribution from the hip muscles increasing during running with load carriage. Specifically, when running without a load, the knee experienced the highest JRF (15.8 BW vs. ankle, 11.3 BW; hip, 12.2 BW) for the subject who completed both walking and running experiments. In contrast, when walking without a load, her ankles were exposed to the highest JRF (4.7 BW vs. knee, 4.4 BW; hip, 4.3 BW). Carrying a 30% BW load increased her JRFs at the hip by 35.2% (ankle, 6.2%; knee, 20.1%) during running, as compared to an increase of 26.2% in her knee JRFs (ankle, 16.4%; hip, 19.0%) during walking [18]. Although this subject ran substantially slower than the others, her running speed (3.0 m/s) was still well above the preferred transition speed between walking and running for nonrunners [2.01 (SD = 0.07) m/s] and runners [2.06 (SD = 0.07) m/s] [41]. Moreover, we observed the same trend in all running participants: the JRF at the knee was greater than that at the ankle or hip under the baseline running condition, whereas the hip JRF was more sensitive and increased markedly as the load carriage increased. The relatively small increase in the peak ankle JRFs in response to load carriage during running reflects the lower degree of the plantarflexor involvement in forward movements when running than when walking [18]. Likewise, the increase in hip JRFs associated with load carriage during running suggests that compared with walking, running requires a relatively greater contribution from the hip extensors to meet the increased mechanical and energetic demands of the leg [42].

For the subject who completed both walking [18] and running experiments, the tibial stress during running was substantially different from that during walking. For the baseline condition, the peak running tibial stress (tension, 90.6 MPa; compression, 136.2 MPa) was more than three times greater than the peak walking tibial stress (tension, 24.1 MPa; compression, 40.3 MPa). Surprisingly, the cumulative stress per cycle for walking (13.6 MPa · s) was 88% of that for running (15.2 MPa · s). A one-time elevation of the peak JRF or tibial stress does not necessarily increase the risk of stress fracture, because elevated mechanical loading is only one factor that contributes to stress-fracture injuries. The total cumulative stress, which is affected by the duration of the stance (i.e., of foot-ground contact time) and is much shorter in running than in walking [43], plays a major role in the biomechanical and biological responses of bone, as do the total time and frequency of training [1].

During BCT, military recruits on average run 36 min and march 129 min a day, depending on their company and initial fitness level [44, 45]. Assuming that a BCT recruit adopts a common step frequency of 60 steps/min for walking and a recommended step frequency of 180 steps/min for running [46], the peak cumulative tibial stress to complete a 36-min running distance reaches 98,496 MPa · s [15.2 (cumulative stress per running cycle) × 180 × 36], and that to complete a 129-min walking distance reaches 105,264 MPa · s [13.6 (cumulative stress per walking cycle) × 60 × 129]. When carrying a 30% BW load under these conditions, the daily cumulative stress due to 36 min of running is 83.3% of that due to 129 min of walking. In other words, the biomechanical impact on the tibia resulting of marching for 129 min is equivalent to that of running for 43 min when the individual carries a 30% BW load. The relatively shorter duration of ground contact in running presumably offsets the effect of the high tibial stress per stride. This is an important finding because, while Service members are not encouraged to run while carrying heavy loads during military training, they are required to complete long-distance tactical foot marches carrying mission-essential equipment (up to 25 kg or 45% BW), which, in essence, leads to the same cumulative stress as a short run with load. If the cumulative effect of internal forces and bone stresses increases the risk of stress-fracture injury, our results suggest that BCT recruits could be more prone to injury when marching an excessive distance than when running a short distance.

One major limitation of the present study stems from the simplifications and assumptions made to construct the model. For example, the knee joint in the current model does not include medial and lateral compartments of joint loads, nor does it fully incorporate complex three-dimensional motions, including translations and rotations. In the absence of medical images necessary for reconstructing subject-specific tibial geometrical structures for FE analyses, we used anthropometric measurements to scale a 50^th^ percentile European female generic musculoskeletal model. Similarly, given that all participants were young and healthy (i.e., ~30 years old), we used tibial material properties mapped from a BMI-matched subject of the same sex for the FE analysis. This approach, along with the use of assumptions regarding material properties (e.g., linear elasticity and isotropy) may affect tibial stress/strain predictions. Furthermore, we chose an effort-based cost function to solve the muscle redundancy problem in the musculoskeletal model. Although this commonly used method predicted patterns of muscle activation qualitatively similar to those of the EMG recordings, it is not suitable for predicting subject-specific muscle activities. This is evident from the discrepancy between the predictions and EMG recordings in the tibialis anterior activation at toe off and rectus femoris activation during early swing (Fig. 2). Lastly, although we quantified the biomechanical alterations during running with load carriage, we only assessed acute effects and did not test for a cause-and-effect relationship between load carriage and stress fracture. Therefore, until more research is conducted in this area, we believe it is premature to identify a correlation between our results and clinically observed injury sites.

## Conclusion

Our study shows how the kinetics and kinematics of the body, as well as bone mechanical stress, adjust over a range of load carriages during running. Our findings highlight the critical role of the hip extensor muscles and their potential injury in women when running with load carriage. More importantly, our results underscore the need to incorporate the cumulative effect of mechanical stress when evaluating injury risk under various exercise conditions. These analyses provide insights into the biomechanical alterations of bone that cannot be captured through traditional force platform measurements. Such findings should serve as a foundation for identifying the mechanical determinants for optimizing military training.

## Abbreviations

BCT: basic combat training
BMI: body mass index
BW: body weight
EMG: electromyography
FE: finite element
GRF: ground reaction force
JRF: joint reaction forces
SD: standard deviation
US: United States
